# Protein unfolding as a switch from self-recognition to high-affinity client binding

**DOI:** 10.1038/ncomms10357

**Published:** 2016-01-20

**Authors:** Bastian Groitl, Scott Horowitz, Karl A. T. Makepeace, Evgeniy V. Petrotchenko, Christoph H. Borchers, Dana Reichmann, James C. A. Bardwell, Ursula Jakob

**Affiliations:** 1 Department of Molecular, Cellular and Developmental Biology, University of Michigan, 830 N-University Avenue, Ann Arbor, Michigan 48109-1048, USA; 2 Howard Hughes Medical Institute, University of Michigan, 830 N-University Avenue, Ann Arbor, Michigan 48109-1048, USA; 3 Department of Biochemistry and Microbiology, Genome BC Proteomics Centre, University of Victoria, 4464 Markham Street #3101, Victoria, British Columbia, Canada V8Z5N3; 4 Present address: Department of Biological Chemistry, Alexander Silberman Institute of Life Sciences, Hebrew University of Jerusalem, Givat Ram Campus, Jerusalem 91904, Israel

## Abstract

Stress-specific activation of the chaperone Hsp33 requires the unfolding of a central linker region. This activation mechanism suggests an intriguing functional relationship between the chaperone's own partial unfolding and its ability to bind other partially folded client proteins. However, identifying where Hsp33 binds its clients has remained a major gap in our understanding of Hsp33's working mechanism. By using site-specific Fluorine-19 nuclear magnetic resonance experiments guided by *in vivo* crosslinking studies, we now reveal that the partial unfolding of Hsp33's linker region facilitates client binding to an amphipathic docking surface on Hsp33. Furthermore, our results provide experimental evidence for the direct involvement of conditionally disordered regions in unfolded protein binding. The observed structural similarities between Hsp33's own metastable linker region and client proteins present a possible model for how Hsp33 uses protein unfolding as a switch from self-recognition to high-affinity client binding.

## Abstract (web summary)

Under stress conditions the molecular chaperone Hsp33 is activated to process unfolded proteins. Here, the authors use *in vivo* and *in vitro* crosslinking and ^19^F-NMR to elucidate the binding site for misfolded proteins and are able to propose a model for its mechanism of action.

## Graphical abstract

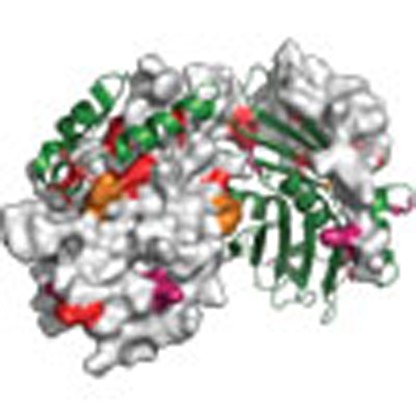

A number of molecular chaperones contain extensive regions of native disorder^1^,^2^. The realization that these disordered regions are often found in regions centric to protein–protein interactions fuelled the idea that these chaperones might engage their non-structured regions directly in client recognition^3^,^4^. Intrinsically disordered structures are characterized by significant flexibility, which may provide a possible explanation for the plasticity that chaperones exhibit in client binding. However, intrinsically disordered regions in proteins also tend to be highly charged^5^,^6^. It therefore remains unclear how these disordered regions in chaperones recognize and bind-folding intermediates, which are characterized by exposed hydrophobic surfaces that are normally buried in the interior of fully folded proteins^7^,^8^,^9^. Unfortunately, experiments addressing these important questions are technically challenging given that both chaperone and client proteins are partially unfolded and thus difficult to structurally characterize. Moreover, promiscuous client binding as is observed with many of these chaperones, often results in only very small chemical shift changes as measured by nuclear magnetic resonance (NMR)^10^.

Over the past few years, a group of stress-specific chaperones have been identified that use post-translational unfolding as the mechanism for their activation^11^. Under non-stress conditions, these proteins are folded and chaperone-inactive. However, upon exposure to specific protein-denaturing conditions, such as hypochlorous acid (HOCl) (for example, Hsp33)^1^, low pH (for example, HdeA)^2^ or elevated temperatures (for example, Hsp26)^12^, the proteins rapidly undergo extensive unfolding and become active as chaperones. These chaperones, appropriately termed conditionally disordered^11^,^13^,^14^, therefore, exist in at least two distinct conformations—a folded inactive conformation and a partially unfolded active conformation^1^,^2^—making them ideal model systems for studying the role of protein disorder in chaperone function.

In this study, we focused on the redox-regulated chaperone Hsp33, which is rapidly activated upon oxidative stress-mediated protein unfolding^15^. Central to Hsp33's activation process is the folding status of a conditionally disordered ∼40-amino-acid linker region (Fig. 1a, light pink), which connects the N-terminal linker-binding domain (Fig. 1a, cyan) with the C-terminal redox switch domain (Fig. 1a, purple). Under reducing conditions, the linker region is stably folded, making extensive contacts with a hydrophobic surface on Hsp33's N-terminal domain (Fig. 1a). Stabilization of the linker domain appears to be further mediated by the redox switch domain, where four absolutely conserved cysteine thiols coordinate zinc with high affinity^15^ (Fig. 1a). Under oxidative stress conditions, disulfide bonds form, zinc is released and the linker region unfolds. This unfolding converts Hsp33 into a high-affinity chaperone that preferentially binds to partially unfolded client proteins^15^,^16^. A single linker-destabilizing mutation constitutively activates Hsp33, providing evidence that linker unfolding is necessary and sufficient for the activation of Hsp33's chaperone function^17^.

To directly investigate the role of conditionally disordered regions in chaperone–client interactions, we used a multipronged approach, combining *in vitro* and *in vivo* crosslinking with site-directed Fluorine-19 NMR (^19^F NMR)/spin-label experiments. We discovered that stress-induced unfolding of Hsp33 exposes a composite amphipathic client-binding site consisting of a stable hydrophobic surface and Hsp33's own conditionally disordered linker region. Structural similarities between Hsp33's own metastable region and the client proteins explain how these chaperones use unfolding to switch from self-recognition to high-affinity client binding.

## Results

### Identification of Hsp33-client interaction sites *in vivo*

To determine the interaction sites between Hsp33 and client proteins in the cellular context, we decided to incorporate the non-canonical amino acid *p*-benzoyl-L-phenylalanine (BPA) into specific locations in Hsp33 and activate it as a zero-length crosslinker via UV irradiation^18^, thereby crosslinking Hsp33 to its client proteins *in vivo*. We reasoned that these experiments could (1) help determine those regions in Hsp33 that come close to its client proteins upon activation of its chaperone function and (2) guide subsequent structural studies *in vitro*.

Wild-type Hsp33 is normally activated by exposing cells to HOCl (ref. 1). However, since HOCl also mediates non-specific protein crosslinking, we decided to introduce BPA into our temperature-regulated variant Hsp33^M172S^ instead of wild-type Hsp33 (ref. 17). Hsp33^M172S^ behaves like wild-type Hsp33 under oxidative unfolding conditions. However, this variant no longer requires HOCl treatment for its activation because of a destabilizing mutation in its N-terminus that causes unfolding of its linker domain and activation of its chaperone function at temperatures as low as 40 °C (ref. 17). This variant, thus, allows us to activate Hsp33's chaperone function *in vivo* by exposing cells to a simple temperature shift and avoids the use of HOCl. Once activated by heat-shock temperatures, the Hsp33^M172S^ variant binds to a wide range of thermally unfolding proteins and sequesters them into insoluble aggregates^17^.

**Figure 1:**
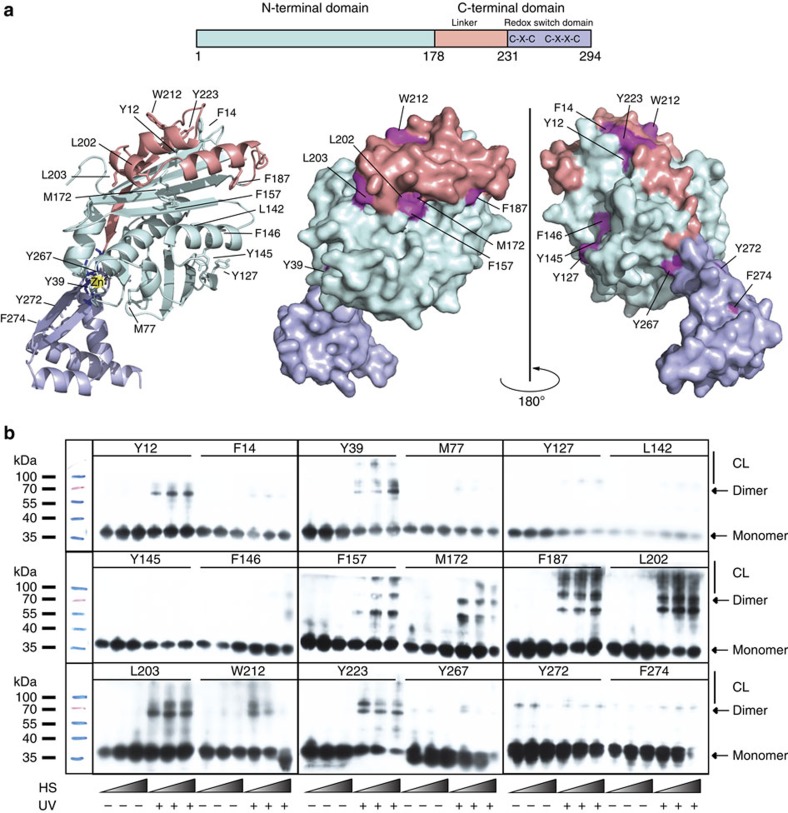
Identification of the Hsp33-client-binding site *in vivo.* (**a**) Eighteen sites in Hsp33 were selected for the incorporation of unnatural amino acids as shown in the I-TASSER-based ribbon (left) and indicated in dark pink on the surface (right) models of reduced *E. coli* Hsp33. Hsp33 consists of an N-terminal domain (cyan), a metastable linker region (light pink) and a redox switch domain (purple), in which four thiolate anions arranged in a C_232_-X-C_234_-X_31_-C_265_-X-Y-C_268_ motif coordinate one zinc ion (red sphere) under reducing conditions. Only one monomer of the dimeric crystal structure is shown. In solution, reduced Hsp33 is monomeric^28^. (**b**) *E. coli* cells overexpressing the Hsp33^M172S-BPA^ variants were shifted from 30 °C to heat-shock conditions (43 °C for 5, 10 or 20 min) (HS). The cells were then either left untreated or exposed to UV irradiation for 10 min to induce crosslinking. Western blot analysis using anti-Hsp33 antibodies was used to visualize the crosslinking products (CL).

Eighteen different sites for BPA incorporation in Hsp33^M172S^ were chosen (Fig. 1a). This selection was primarily based on the rationale that the substitution of the phenylalanine-like BPA for an existing Phe or a residue that was found substituted for Phe in at least one known Hsp33 homologue would be unlikely to substantially affect Hsp33's tertiary structure. We individually replaced the selected codons with an amber (TAG) stop codon and co-expressed the respective Hsp33^TAG^-containing plasmids together with the pEVOL single-vector construct, which contains two copies of *Methanocaldococcus jannaschii* aminoacyl-tRNA synthetases and an optimized suppressor tRNA^CUA^, using a Hsp33-deletion mutant of *Escherichia coli*^19^. All mutant strains expressed full-length Hsp33 in a BPA-dependent manner (Supplementary Fig. 1a).

We exposed live cells that expressed the BPA-substituted Hsp33 variants to UV irradiation at either 30 °C (Supplementary Fig. 1b) or for various times after shift to heat-shock temperatures (Fig. 1b). Subsequent western blot analyses of whole-cell extracts using antibodies against Hsp33 were used to monitor which proteins crosslink with Hsp33 and to what extent. As depicted in Fig. 1b and summarized in Supplementary Table 1, we found the largest extent of Hsp33 crosslinking species with mutant variants that contained BPA in or adjacent to the flexible linker region (F157, M172, F187, L202, L203, W212 and Y223). Modest amounts of crosslinking products were found at some additional sites in the N-terminus (Y12 and Y39), whereas most of the remaining BPA incorporation sites did not undergo any substantial crosslinking (Supplementary Table 1). To test whether the observed higher migrating bands are indeed crosslinking products between Hsp33 and client proteins, or represent crosslinks between Hsp33 molecules in higher oligomeric states, we purified two of our Bpa mutant variants, Hsp33^M172SY145BPA^ (negative in *in vivo* crosslinking experiments) and Hsp33^M172SL202BPA^ (positive in *in vivo* crosslinking experiments). *In vitro* crosslinking experiments revealed no additional bands apart from Hsp33 dimers migrating at ∼70 kDa (Supplementary Fig. 1c), strongly suggesting that the higher migrating bands that we observed in our *in vivo* crosslinking studies do indeed represent crosslinks between activated Hsp33 and cellular client proteins.

Note that most of the BPA mutant variants that crosslinked at all showed also a discrete crosslinking product, whose size corresponds with that of the crosslinked Hsp33 dimer (Fig. 1b; Supplementary Fig. 1c). As was the case with all other crosslinking species, this band only became detectable upon UV crosslinking (Fig. 1b and Supplementary Fig. 1b). This result was intriguing since, based on the available crystal structures of Hsp33 (PDB 1HW7), these sites are far away from each other, making crosslinking quite unlikely. The only published Hsp33 structure that places these crosslinked sites into close vicinity is the structure of an oxidized Hsp33^1-255^ fragment (PDB 1HW7), in which the linker region of one subunit folds on top of the N-terminal linker-binding domain of the second subunit^20^ (Supplementary Fig. 1d). This domain-swapped conformation could explain most of the Hsp33-Hsp33 crosslinks that we observed *in vivo.* That the same Hsp33-BPA substitutions could crosslink either with each other or with client proteins suggests that the sites that Hsp33 uses for client binding under oxidative stress conditions overlap with those engaged in interactions between Hsp33's N-terminus and the linker region under non-stress conditions. Alternatively, Hsp33 crosslinks might be mediated by extended flexible loops coming transiently into sufficiently close vicinity. No substantial inter-Hsp33 or inter-client crosslinking bands were observed for Hsp33 variants containing BPA in a region of the N-terminus that lies opposite from the linker-binding surface (Y127, L142, Y145 and F146), or in the C-terminal redox switch domain (Y267, Y272 and F274). This finding agrees well with previous results, which indicated that the C-terminal redox switch domain has a primarily regulatory role and is not involved in substrate binding^16^. These results provided a first clue as to the location of the client-binding site in Hsp33 and suggested that both the hydrophobic linker-binding surface as well as the flexible linker region might become directly involved in client binding following activation.

### Monitoring conformational changes in Hsp33 by ^19^F NMR

Our *in vivo* crosslinking results suggested that Hsp33 can interact both with client proteins as well as with itself during heat-shock conditions *in vivo*. Crosslinking, however, is sensitive to regions of flexibility, non-equilibrium conditions and residue chemistry. To address these issues, we decided to use an independent approach to evaluate the interaction status of the residues we found using *in vivo* crosslinking. The strategy we employed is a novel application of *in vitro*^19^F NMR spectroscopy. We reasoned that by utilizing several of the same amber stop codon positions that we used for *in vivo* crosslinking, we could instead incorporate 4-(trifluoromethoxy)-phenylalanine (tFPA) (refs 21, 22, 23) at our preselected Hsp33 sites. Upon purification of these mutant variants, we would then be able to directly detect client binding by monitoring the ^19^F NMR signal at these selected sites. ^19^F NMR provides several advantages; ^19^F is the natural isotope of fluorine and thus is present at 100% natural abundance, features high chemical shift dispersion and is one of the most sensitive NMR nuclei available in nature, with a sensitivity of 83% compared with ^1^H, the most widely used isotope in protein NMR^24^. Furthermore, ^19^F is a strong reporter on changes in local chemical environments, including solvent exposure^25^. These features, combined with the mobility of the trifluoromethoxy-group, make tFPA an extraordinarily sensitive chemical shift probe for monitoring binding or conformational changes^26^,^27^. Most importantly, because of the site-selective nature of the amber stop codon strategy, there are no ambiguities in resonance assignment, as only one amino acid is labelled at a time, and it does not suffer from the same ambiguities as crosslinking arising from reactivity constraints. This combination of advantageous traits should make this method particularly suitable for disordered proteins, whose NMR spectra are typically extremely challenging to interpret^28^ (Supplementary Fig. 2a,b). Use of a simple paramagnetic relaxation enhancement (PRE) experiment with our Hsp33-tFPA variants (see below for details) should be able to distinguish direct binding sites from areas of conformational changes within Hsp33.

**Figure 2:**
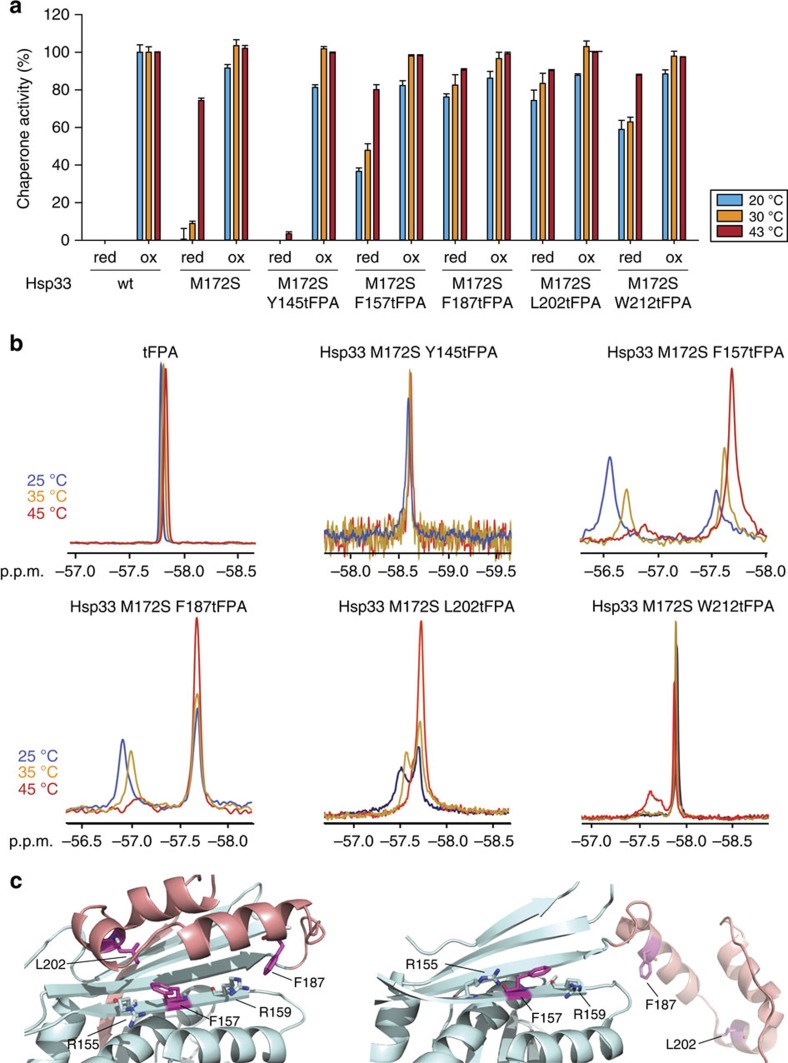
Monitoring conformational rearrangements in purified Hsp33^M172-tFPA^ variants using ^19^F NMR. (**a**) Chaperone activity of reduced, zinc-reconstituted or HOCl-activated wild-type Hsp33, Hsp33^M172S^ or Hsp33^M172S-tFPA^ variants. Chaperone activity was measured by testing the influence of a four-fold molar excess of Hsp33 on the aggregation of chemically unfolded CS at either 20 °C (blue bars) or 30 °C (orange bars) or on thermally unfolded CS at 43 °C (red bars). Chaperone activity of 0% is defined as the light-scattering signal 4 min after addition of CS in the absence of chaperones. Activity of 100% corresponds to the light-scattering signal of CS in the presence of a four-fold molar excess of wild-type Hsp33 that had been activated for 2 min in 200 μM HOCl at 30 °C. All experiments were conducted at least 3–5 times and the s.e.m. is shown. (**b**) Temperature dependence of the ^19^F NMR signal in select Hsp33^M172S-tFPA^ mutants. ^19^F NMR spectra of tFPA alone or the indicated mutant variants were recorded at either 25 °C (blue), 35 °C (orange) or 45 °C (red). (**c**) N-terminal linker-docking surface (cyan) and the metastable linker region (pink) of *E. coli* Hsp33 in the inactive, closed state (left) (I-TASSER model) and in the activated, open state (right) (PDB 1HW7). The close proximity of F157 and F187 (and L202) to Arg155 and Arg159 in the closed state of Hsp33 is likely responsible for the distinctive down-field chemical shift change observed in the mutant variants under inactivating conditions.

We decided to focus on clearly positive *in vivo* crosslinking sites (Y12, Y39, F157, F187 and L202), one negative site (Y145) and one ambiguous site (W212) (Fig. 1b). We successfully purified all of the proteins except Hsp33 variants harbouring non-natural amino acids at either position Y12 or Y39. These two protein variants could not be expressed in sufficiently high quantities. We then tested the purified proteins for their *in vitro* chaperone function under both reducing and oxidizing conditions (Fig. 2a). All of the tested proteins showed wild-type-like activity when oxidized and disulfide bonded, indicating that client binding was not substantially affected by the introduction of tFPA at these sites. Moreover, although the activity of the reduced tFPA mutant variants was generally higher at 30 °C than the parental Hsp33^M172S^ mutant protein, all but one of our mutant proteins (Hsp33^M172SY145tFPA^) maintained some degree of its temperature-induced chaperone activation. Their higher than parental activity at 30 °C suggests that the tFPA substitutions act to further destabilize the linker region. This idea is consistent with the finding that most of our crosslinking-positive Hsp33-BPA variants also showed some self-crosslinking in addition to client protein crosslinking at 30 °C (Supplementary Fig. 1b). One mutant variant, Hsp33^M172SY145tFPA^ was chaperone-inactive at both temperatures. This result agrees well with our *in vivo* data, where we did not identify any crosslinking products at either 30 or 43 °C, despite the fact that the chaperone is active when oxidized (Fig. 1b and Supplementary Fig. 1b). This finding suggested that the Y145tFPA mutation suppresses the temperature regulation of the Hsp33^M172S^ mutant variant.

Only two of our mutant variants, Y145tFPA and W212tFPA, revealed a single peak in the ^19^F NMR spectrum at 25 °C, as would be expected from a single labelled residue in a homogeneous environment (Fig. 2b). The three other variants, however, displayed two resonances in their 1D–^19^F NMR spectrum. These results strongly suggested that the three fluorinated residues, which reside in the linker-region docking surface of Hsp33's N-terminus (F157) or the linker region (F187 and L202), exist in at least two chemical environments that interchange slowly on the NMR timescale (millisecond exchange rate or slower). For all three variants, we found that the chemical shift of the up-field peaks was similar to that of the unbound tFPA (Fig. 2b). The simplest interpretation of these results is that the up-field peak corresponds to a conformation in which the tFPA is surface exposed, whereas the down-field peak corresponds to a more buried chemical environment (Fig. 2b). We thus hypothesized that the respective up-field resonances represent the unfolded, chaperone-active state of Hsp33, and the down-field peaks represent the folded, chaperone-inactive state of Hsp33. This hypothesis was further supported by quantum mechanics (QM) chemical shift calculations, which allowed us to quantify the effects of neighbouring residues on the chemical shifts. QM calculations demonstrated that small changes in the environment or the bond angle of our tFPA probe would result in measurable differences for the chemical shift (Supplementary Fig. 2c,d). These calculations revealed that in the case of F157 and F187, the protein interior indeed causes a down-field chemical shift change because of the close proximity to the side chains of R155 and R159 in the folded state (Fig. 2c, left). We tested this hypothesis by monitoring the ^19^F NMR signals of F157tFPA and F187tFPA as a function of temperature and found that the area of the putative active-state peak indeed reversibly increased in size with increasing temperature and unfolding, whereas the area of the putative inactive state decreased (Fig. 2b, compare blue and red traces). No change in peak area or height upon shift to higher temperatures was detected for unbound tFPA. These results are in excellent agreement with our previous results^17^, which showed that the linker region in Hsp33^M172S^ unfolds in a temperature-dependent manner, leading to the activation of the protein. Thus, we concluded that for both F157 and F187, the up-field resonance peak represents the active Hsp33 chaperone with the linker region freed from the surface of Hsp33, and the down-field peak represents the closed, inactive state of Hsp33, in which the binding site is closed off from solution and brought in immediate contact with R155 and R159. For L202, two peaks were also detected in the NMR spectrum that changed size with temperature, potentially also representing the active, open state and the closed, inactive state, similar to F157 and F187. In contrast, increasing the temperature of the W212-tFPA mutant variant led to the appearance of an additional down-field resonance peak, which could potentially represent a second active chemical environment (Fig. 2b).

### Monitoring Hsp33-client interactions *in vitro*

**Figure 3:**
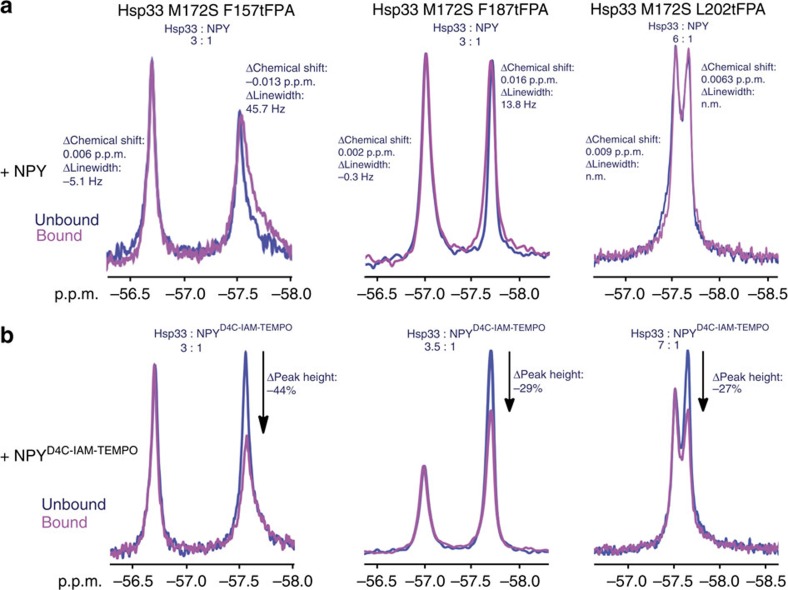
Monitoring Hsp33-client-binding interactions using site-specific ^19^F NMR. ^19^F NMR spectra of select Hsp33^M172StFPA^ variants in the absence (blue) or presence (magenta) of (**a**) NPY or (**b**) NPY labelled with the paramagnetic spin-label TEMPO. The incubation temperature was set to 35 °C. All experiments were conducted at least three times.

With the active and inactive states of Hsp33 identified using our tFPA substitutions, we proceeded to test how client binding affects the respective resonances in the ^19^F NMR spectra. For these experiments, we used the soluble client peptide neuropeptide Y (SNPY) as a Hsp33 substrate^16^ (Supplementary Fig. 3a). Changes in chemical shift or line broadening upon addition of neuropeptide Y (NPY) would indicate differences in the local environment of tFPA-labelled residues or in the dynamics of the protein, respectively. Upon titrating NPY into a solution containing the ^19^F-labelled chaperone variants, we observed distinct chemical shift changes and increased line broadening for the up-field active-state peaks of both F157 and F187 (Fig. 3a). In contrast, the inactive states (that is, down-field peaks) displayed comparatively little to no line broadening or chemical shift changes, consistent with the idea that clients bind to the more unfolded, active state of Hsp33. Furthermore, upon addition of NPY, the peak area of the presumed inactive state of both F157 and F187 slightly decreased, whereas the peak size of the presumed active state increased, indicating that the addition of the chaperone substrate NPY shifts the equilibrium in the direction of active client-bound Hsp33. The Y145tFPA variant displayed no chemical shift or linewidth changes upon addition of NPY (Supplementary Fig. 3b), consistent with the finding that this mutant variant is inactive under these conditions (Supplementary Fig. 3a). Instead, upon addition of NPY, we observed significant precipitation of this mutant variant, which likely explains the decrease in peak size (Supplementary Fig. 3b). The peaks for the L202tFPA (Fig. 3a) and W212tFPA (Supplementary Fig. 3b) variants displayed only very small chemical shift changes and no detectable linewidth changes, leaving their role in binding still unclear.

To directly identify whether L202tFPA and W212tFPA are at client-binding sites, and to confirm that F157 and F187 indeed represent areas of direct client binding and do not instead show chemical changes and line broadening just because of conformational rearrangements in Hsp33, we employed a NPY analogue (NPY^D4C^) labelled with a PRE tag. PRE tags line-broaden residues that are close in space to the tag, affecting residues in a distance-dependent fashion^29^. For this purpose, we chose 4-(2-iodoacetamido)-TEMPO (IAM-TEMPO), which we conjugated to an engineered cysteine residue in NPY (NPY^D4C^). The PRE tag did not substantially affect binding of NPY to our mutant Hsp33 variants, as tested by competition assays (Supplementary Fig. 3a). To evaluate the line-broadening effect of the PRE tag, we measured the relative peak-height change of the Hsp33-tFPA signals upon binding NPY^D4C-IAM-TEMPO^ versus binding unlabelled NPY. Analysis of peak heights is equivalent to evaluating line broadening as long as the peaks are properly normalized, and is commonly used in biomolecular NMR analysis of PREs. Notably, we found that addition of sub-stoichiometric concentrations of NPY^D4C-IAM-TEMPO^ to either Hsp33-F157tFPA or Hsp33-F187tFPA caused a reproducible loss in peak height of only the active-state resonances for each of the residues, implicating F157 and F187 as sites of direct client binding (Fig. 3b, Supplementary Fig. 3d,f). No pronounced change in the ratio between the active or inactive peak was observed when buffer alone was added to the proteins (Supplementary Fig. 3e), while titration of the NPY^D4C-IAM-TEMPO^ peptide revealed a peptide-concentration-dependent decrease in the peak height of the active-state resonance (Supplementary Fig. 3f). Using the extent of PRE line broadening and the stoichiometry of binding (Fig. 3b)^30^ we estimated an upper bound for the average interaction distance between NPY and residues F157 and F187 of ∼13–15 Å. Similar levels of PRE line broadening (that is, decrease in peak height) were also observed for the presumed active-state peak of Hsp33-L202tFPA (Fig. 3b), whereas the Y145tFPA and W212tFPA substitutions did not reveal any line broadening upon addition of NPY^D4C-IAM-TEMPO^, indicating that these residues are not directly involved in substrate interaction (Supplementary Fig. 3c). These results show that by using unnatural amino acid substitutions followed by *in vivo* crosslinking, ^19^F NMR, and spin-label experiments, we obtained valuable insights into Hsp33's activation and client-binding process, and identified at least three sites in Hsp33 that are indeed bona fide client interaction sites—one in the linker-docking surface (F157) and two directly in the linker region of Hsp33 (F187 and L202).

### *In vitro* crosslinking reveals client-binding sites in Hsp33

**Figure 4:**
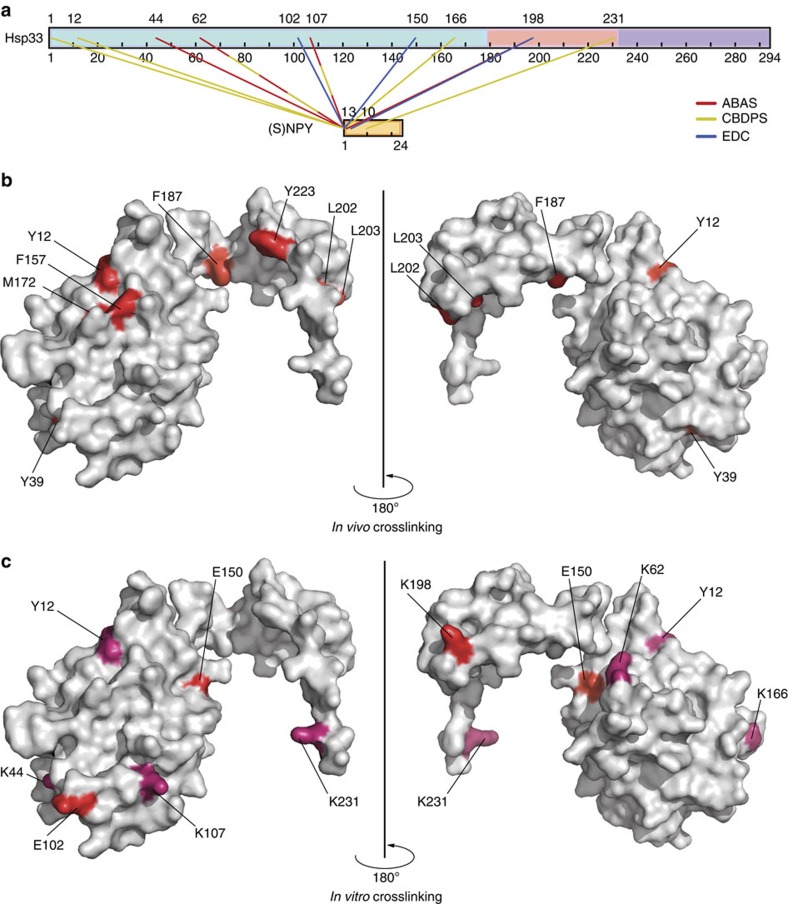
Determining the Hsp33-client interaction sites using *in vitro* crosslinking. (**a**) *In vitro* crosslinks specifically between oxidized (activated) wild-type Hsp33 and (S)NPY-peptide are displayed in a linear representation created with Cross-Link Viewer^53^ (SNPY is the NPY peptide containing one additional serine residue at the N-terminus). Residues that crosslinked with more than one crosslinker are shown as dashed lines in the respective colours (ABAS: red; CDBPS: yellow; and EDC: blue). (**b**) *In vivo* and (**c**) *in vitro* crosslinking sites are indicated on the crystal structure of oxidized, domain-swapped *E. coli* Hsp33^1-255^ (PDB 1HW7). Only one of the two subunits that are found in the crystal structure is shown. Zero-length *in vitro* crosslinks with EDC are indicated in red; long- and medium-range crosslinks (CBDPS and ABAS) are shown in dark pink.

**Figure 5:**
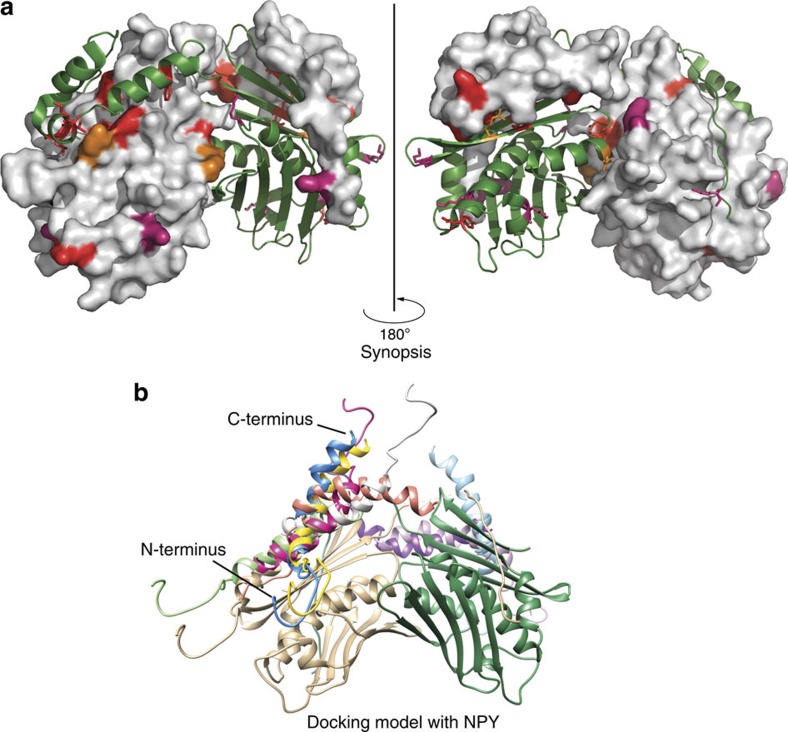
The client-binding site in Hsp33. (**a**) Structure of domain-swapped *E. coli* Hsp33^1-255^ (PDB 1HW7) with one monomer in surface representation and the other one in cartoon depiction. All *in vivo* crosslinking-positive residues, all zero-length *in vitro* crosslinking sites, and ^19^F NMR-positive sites are highlighted in red. Long- and medium-range *in vitro* crosslinking sites are marked in dark pink, and sites previously suggested to be involved in client binding by limited proteolysis experiments^16^ are depicted in orange. Most identified client interaction sites overlap with interaction sites between Hsp33's linker-docking region and the linker region. (**b**) Docking model of NPY peptide (PDB 1RON) using the truncated oxidized Hsp33 model (residues 182–219 were removed from PDB 1HW7). The top ten docking models are shown in various colours. The Hsp33 subunits are depicted in green and gold, respectively.

The number and identity of residues that are chosen for substitution in a protein clearly limit the scope of *in vivo* crosslinking studies. To use an alternative approach that does not rely on amino acid substitution choices, we therefore conducted *in vitro* crosslinking studies followed by mass spectrometric analysis. We used crosslinking reagents that vary in their spacer distance (14, 7 and 0 Å) and performed crosslinking experiments with reduced inactive or oxidized active wild-type Hsp33 using NPY as the client peptide (Supplementary Fig. 4a). Digestion of the protein mixtures with either protease K or trypsin combined with tandem mass spectrometric (MS/MS) analysis was used to identify the crosslinking products. To facilitate the identification of Hsp33-NPY crosslinked peptides, we either used isotopically coded crosslinkers, such as collision-induced dissociation-cleavable amine-reactive 14 Å-length cyanur-biotin-dimercapto-propionyl-succinimide (CBDPS-H_8_/D_8_; ref. 31) or photoreactive 7 Å-length azido-benzoic-acid-succinimide (ABAS-^12^C_6_/^13^C_6_; ref. 32), or worked with an equimolar mixture of ^14^N- and ^15^N-metabolically labelled Hsp33 in the case of the zero-length crosslinker (3-dimethylaminopropyl)carbo diimide (EDC) (flow chart shown in Supplementary Fig. 4b). Analysis of the crosslinking products by MS/MS analysis revealed a number of crosslinks between activated Hsp33_ox_ and NPY, which we did not observe when we tested inactive Hsp33_red_ (see Supplementary Data for the complete list of identified *in vitro* crosslinking products and MS/MS analyses). Several of the amino acids in Hsp33_ox_ that we found to crosslink with NPY *in vitro* were independently identified using at least two different crosslinkers (Fig. 4a and Table 1, in bold). Residues Y12, K44, K62 and K198 are of special interest since they were either identified as *in vivo* crosslinking sites in this study (Fig. 4b,c), or positively identified as residues in Hsp33_ox_ that are more protected from tryptic digest when in complex with thermally unfolded luciferase^16^ (Fig. 5a). Moreover, K198, which is located in the linker region between Y187 and L202, was found to crosslink with the zero-length crosslinker EDC (Fig. 4c). This finding indicates a very close proximity between Hsp33's linker region and its client peptide, NPY. Most other crosslinks between Hsp33_ox_ and NPY were found with residues of the linker-docking surface of the N-terminal domain (Fig. 4b,c). Taken together, these experiments provide independent evidence that the chaperone Hsp33 uses residues of its linker-docking region as well as its natively disordered linker region to directly bind its unfolded client proteins. To verify that the linker-docking region indeed provides a favourable surface for the binding of client proteins, we performed protein–protein docking simulations, in which the linker region of Hsp33 was removed, exposing the docking site to solvent (Fig. 5b). Notably, using a blind docking procedure in which no experimental or computational constraints were added to identify the binding site, all of the top 10 docking hits localized NPY to this linker-docking region. This docking simulation supports our ^19^F NMR-binding data that the linker-docking region provides a favourable surface for binding partially folded substrates.

## Discussion

A common feature of a growing group of chaperones, such as Hsp33, HdeA/HdeB and small heat-shock proteins, is their ability to specifically activate their chaperone function through stress-induced conformational rearrangements, unfolding and/or changes in oligomerization states^2^,^12^,^33^. By using these mechanisms, these so-called stress-specific chaperones are activated only under stress conditions that cause protein unfolding and thus demand the presence of an additional chaperone activity^11^. Once in their activated state, these chaperones bind potently and non-discriminatingly to a large number of protein folding intermediates. Recent evidence suggested that the metastable regions, whose folding status is central to their activation process, do not simply constitute molecular gates that control accessibility to potentially hydrophobic client-binding sites^2^,^28^,^33^, but participate in the binding of unfolding client proteins. However, structural studies that would allow us and others to unambiguously address these questions rely on homogeneous and highly stable protein complexes, and require participants that are folded and well-behaved. In the case of conditionally disordered chaperone–client complexes, both chaperones and client proteins are at least partially unstructured, and the folding status of neither partner is well-defined in the bound state. Methodologies that have been used in the past to track down client protein-binding sites in chaperones involve limited proteolysis and hydrogen/deuterium (H/D) exchange measurements combined with MS/MS analysis^16^,^34^,^35^. With these approaches, changes in the accessibility of proteolytic sites or degrees/rates of H/D exchange in the absence and presence of client proteins are used as an indication of conformational changes in the chaperone that occur upon client binding. These studies, although very powerful in identifying sites of major conformational disturbance, do not distinguish between direct binding events and structural changes that take place in response to the client binding somewhere else in the protein. This limitation became apparent in recent studies on Hsp33, in which client-induced folding and unfolding events were observed in the linker region of Hsp33 but evidence for direct interactions between client proteins and the conditionally disordered linker region were missing^16^.

To address this question, which indeed would appear to pertain to many known chaperone–client protein complexes, we decided to employ a novel approach, incorporating unnatural amino acids into Hsp33 to perform both *in vivo* crosslinking and ^19^F NMR, using the same amber stop codon mutations. This method, followed by unbiased *in vitro* crosslinking experiments, unequivocally revealed a composite client-binding site in Hsp33 that consists of polar residues from the flexible linker region and nonpolar residues from the N-terminal linker-docking surface of Hsp33 (Fig. 5a). These results agreed in part with our previous limited proteolysis and H/D exchange studies, which demonstrated that client binding to activated Hsp33 limits access of proteolytic sites and significantly affects the thermodynamic stability of the linker region, suggesting that the linker region is involved in client binding^16^. In contrast, however, our previous study provided very little evidence for the involvement of the hydrophobic linker-binding platform in client binding, whereas the crosslinking experiments conducted in this study unequivocally pointed to a substantial involvement of this region in Hsp33-client interactions. The reason for this apparent discrepancy is likely found in the technical limitations of our previous techniques, including the absence of testable proteolytic cleavage sites in the hydrophobic linker-binding platform, and the possibility that H/D exchange measurements lack the necessary sensitivity to detect some of these interactions. In either case, our results that Hsp33 uses a composite binding site composed of a flexible polar linker region and a stable hydrophobic binding surface, are highly reminiscent of recent reports on the mechanism of substrate recognition by the canonical chaperone TRiC/CCT (ref. 36). In their study, the authors postulate that charged-charged interactions between the chaperonin and its client protein confer specificity to binding, while nonpolar contacts allow for tight packing and additional stabilization of the complex. In analogy to this mechanism, it is tempting to speculate that Hsp33 uses its polar and highly flexible linker region to undergo initial electrostatic interactions with client proteins, thereby positioning and guiding them to the nonpolar N-terminal surface for additional hydrophobic interactions and increased stability. Subsequent rearrangement and refolding of the linker region^16^ might further contribute to the stabilization of the complex. These results suggest that unfolded protein recognition by chaperones might follow principles that go way beyond simple hydrophobicity, a force that has up to now been thought to drive most chaperone–client interactions^37^.

Our studies suggest that Hsp33 uses many of the same interactions between its linker region and the linker-docking domain under reducing, non-stress conditions to engage with partially folded client proteins under oxidizing, activating conditions. These results could explain why activated Hsp33 binds preferentially to client proteins with substantial amounts of secondary structure elements^16^, since they could resemble the conformation of Hsp33's own linker region under reducing conditions. Intriguingly, the flexible linker region in the oxidized Hsp33 dimer shows partial helicity, similar to the peptide substrate model, NPY, both when free in solution and in the docking model. The similarity between these two sets of conformations and their binding modes to Hsp33 strongly suggests that the domain swap observed in oxidized Hsp33 dimers in the absence of client proteins recapitulates substrate binding. Furthermore, Hsp33's clients bind both the flexible linker as well as its docking platform, suggesting a potential model for how Hsp33 can effectively transfer partially folded clients to foldases such as the DnaK/DnaJ/GrpE system through multiple partially overlapping binding sites extending away from Hsp33's surface. As such, exposed hydrophobic residues of the unfolded protein could be protected by the docking platform, but then transit the flexible linker to the waiting refolding system.

These results uncover the power of the amber stop codon suppression approach to untangle difficult heterogeneous or dynamic experimental systems. The site-labelling strategy presented here combines *in vivo* and *in vitro* probing methods in a generalizable approach. This strategy could conceivably unravel many such experimentally difficult binding interactions in conditionally folded or partially folded proteins, where traditional techniques cannot overcome the complex barriers posed by the underlying biology.

## Methods

### Strains and plasmids

The incorporation of amber (TAG) stop codons at individual preselected sites in Hsp33^M172S^ was performed by site-directed mutagenesis using pET11a *hslO*^*M172S*^ as template^17^. After sequencing the plasmids, the inserts were recloned into respective pET21b to generate His-tagged versions of the Hsp33^*M172S*^ variants. All plasmids were transformed into the *E. coli* expression strain BL21 *hslO*^*−*^. A complete list of strains and plasmids used in this study can be found in Supplementary Table 2.

### *In vivo* crosslinking

pET11a plasmids containing the respective Hsp33^M172S^-amber mutants were co-transformed with pEVOL (ref. 19), which contains two copies of *M. jannaschii* aminoacyl-tRNA synthetases and an optimized suppressor tRNA^CUA^, into BL21 Δ*hslO* (a Hsp33-deleted mutant) of *E. coli*^19^. We then grew the strains in the absence and presence of BPA and confirmed that full-length Hsp33 mutant variants were only produced when BPA was supplied to the media (Supplementary Fig. 1a). These strains were plated on lysogeny broth (LB) plates supplemented with 200 mg ml^−1^ ampicillin and 34 mg ml^−1^ chloramphenicol. The next day, transformants were scraped off the plates and used to inoculate 5 ml of LB medium supplemented with 1 mM ZnCl_2_, 1 mM BPA (Bachem Holding AG), 200 mg ml^−1^ ampicillin and 34 mg ml^−1^ chloramphenicol to an OD_600_ of 0.4 (ref. 38). After 1 h of incubation at 37 °C, cells were shifted to 30 °C and protein expression was induced with 0.2 mM IPTG and 0.2% L-arabinose. Cells were harvested after 6 h, washed twice and resuspended in phosphate-buffered saline, pH 7.4 using 100 μl of phosphate-buffered saline per 1 OD_600_ unit. Cells were then incubated at either 30 °C or heat-shock temperature (43 °C) for the indicated times with shaking (400 r.p.m.). Immediately after incubation, 100 μl cells were transferred into 96-well plates and placed on ice. The samples were crosslinked for 10 min using a 25 W, 365 nm UV lamp (UVGL-58; 115 V, 60 Hz, 0.16 A). UV irradiation was applied from the top, using a distance of ∼2 cm from the 96-well plates. After UV crosslinking, cells were lysed with lysozyme (50 mg ml^−1^), followed by sonication (15%, 15 s) and DNase treatment (1 mg ml^−1^; ref. 39). Finally, samples were supplemented with × 5 reducing SDS Laemmli buffer, separated on a 12% SDS–polyacrylamide gel electrophoresis gel, and Hsp33 was visualized by western blot using polyclonal antibodies against Hsp33 in a 1:5,000 dilution^1^,^40^. Select Hsp33-Bpa variants were purified using the purification protocol described below.

### *In vivo* incorporation of 4-trifluoromethoxy-phenylalanine

pET21b plasmids containing a His-tagged version of the respective Hsp33^M172S^-amber variants were co-transformed with pDule2-pCNF (plasmid encoding an orthogonal aminoacyl-tRNA synthetase/tRNA pair)^27^ into BL21 *hslO*^*−*^. The transformants were grown overnight on LB plates, supplemented with 200 mg ml^−1^ ampicillin and 100 mg ml^−1^ spectinomycin. Freshly transformed cells were scraped off the plates and used to inoculate 1 l of protein expression medium (12 g l^−1^ tryptone, 24 g l^−1^ yeast extract, 4% glycerol (v/v), 2.1 g l^−1^ potassium phosphate (monobasic) and 12.5 g l^−1^ potassium phosphate dibasic), supplemented with 1 mM ZnCl_2_, 1 mM tFPA (JRD Fluorochemicals), 200 mg ml^−1^ ampicillin and 100 mg ml^−1^ spectinomycin to an OD_600_ of 0.4. After 1 h of incubation at 37 °C with shaking (200 r.p.m.), cells were shifted to 20 °C and proteins were induced with 0.2 mM IPTG and 0.2% (w/v) L-arabinose. Cultures were harvested the next day by centrifugation (4,000*g*, 20 min, 4 °C). Cells were resuspended in 40 mM potassium phosphate (KPi), 200 mM KCl, 10 mM imidazole and pH 7.5 supplemented with one tablet of protease inhibitor (Roche) and 1 mM phenylmethylsulphonyl fluoride (Sigma-Aldrich). Cell lysis was performed using a French press cell (3 × 1,400 p.s.i.). The cleared lysate was applied onto a nickel-NTA column (GE Healthcare) equilibrated in 40 mM KPi, 200 mM KCl, 10 mM imidazole, pH 7.5 and Hsp33 variants were eluted using a gradient from 10 to 250 mM imidazole in the same buffer. For further purification of the His-tagged Hsp33 variants, anion-exchange chromatography (Q-sepharose HP, GE Healthcare) was used. The column was equilibrated with 40 mM KPi, 200 mM KCl and Hsp33 was eluted using a gradient from 200 to 700 mM KCl in 40 mM KPi, pH 7.0. Purified protein fractions were pooled, dialyzed against storage buffer (40 mM KPi, pH 7.5) at 4 °C, concentrated using Amicon Ultra-15 centrifugal tubes (EMD Millipore) and stored at −80 °C.

### Hsp33 purification

Unlabelled wild-type Hsp33 was expressed and purified^41^. In brief, Hsp33-expressing cells (BL21, pET11a, *hslO*) were grown at 37 °C in the presence of 1 mM ZnCl_2_ and harvested 6 h after induction with 1 mM IPTG. Cells were resuspended in buffer A (40 mM HEPES-KOH, 0.2 M KCl (pH 7.5), one tablet Roche complete protease inhibitor mix, 2 mM phenylmethylsulphonyl fluoride) and lysed (French Press, two cycles, 14,000 p.s.i.). The cleared supernatant was applied onto a Q-Sepharose column (Pharmacia) and eluted with a KCl gradient between 450 and 600 mM KCl in buffer A. The Hsp33-containing fractions were dialysed against 10 mM potassium phosphate (pH 6.8) buffer and applied onto a hydroxylapatite column. Hsp33 eluted between 10 and 70 mM potassium phosphate. The Hsp33-containing fractions were then loaded onto a Superdex 75 (Pharmacia) equilibrated in buffer A. Highly purified Hsp33 fractions from the Superdex column were dialyzed against storage buffer (40 mM potassium phosphate buffer (pH 7.5)).^15^N-labelled wild-type Hsp33 and Hsp33-Y12E were expressed in M9 minimal medium supplemented with 0.1% (w/v) ^15^N-labelled ammonium chloride and purified as before.

### Preparation of NPY^D4C^ IAM-TEMPO

NPY^D4C^-peptide (GenScript) was dissolved in 40 mM HEPES, pH 7.5 and its concentration was determined at 280 nm (*ɛ*=5,960 M^−1^ cm^−1^). A 10-fold molar excess of tris(2-carboxyethyl)phosphine (TCEP) was added and incubated for 30 min at 30 °C with shaking (400 r.p.m.) to fully reduce NPY^D4C^. Then, 4-(2-Iodoacetamido)-2,2,6,6-tetramethyl-1-piperidinyloxy (4–2-(iodoacetamido)-TEMPO or IAM-TEMPO, Sigma-Aldrich) was added in a stepwise manner (1/5 of the total volume added every minute) to yield a final molar ratio of peptide to label of 1:10 (ref. 42). This solution was incubated in the dark for 2 h at 30 °C with shaking (400 r.p.m.), followed by a dialysis against 40 mM KPi, pH 7.5 using a Spectra/Por Micro Float-A-Lyzer device (500 Da–1 kDa) (Spectrum Laboratories) to remove residual, non-reacted IAM-TEMPO. The concentration of NPY^D4C^ IAM-TEMPO was determined before use in NMR experiments.

### Computational methods

Full-length models of Hsp33 for docking procedures and QM calculations were produced using I-TASSER (ref. 43) and ModRefiner (ref. 44). Docking models were generated by removing residues 182–219 from the Hsp33 model (PDB 1HW7), followed by Z-DOCK automated protein–protein docking^45^ between the Hsp33 variant and the conformational ensemble of NPY (ref. 46) with no added constraints. tFPA-containing models for QM calculations were generated by mutating in tFPA into residue 187 in the I-TASSER Hsp33 model in UCSF Chimera 1.10 (ref. 47), followed by structure minimization using the default parameters. Residues were then trimmed to a minimal model surrounding tFPA, and hydrogens added to neutralize the system in Gaussview 5 (Gaussian). The tFPA side chain orientation was then optimized while holding the remainder of the structure constant using wb97 × d/6–31+G(d,p) with implicit water solvation, followed by chemical shift calculation using gauge-independent atomic orbitals with the same calculation parameters in Gaussian 09 (ref. 48). The structure of trifluoroacetic acid was optimized by the same methods for chemical shift referencing, as were the calculations of chemical shifts and conformational energies for tFPA alone.

### Hsp33 chaperone activity and competition assay

Preparation of reduced, inactive Hsp33 (Hsp33_red_) or HOCl-oxidized active Hsp33 (Hsp33_ox_), and chaperone-activity measurements using either chemically or thermally denatured citrate synthase (CS) as client protein^16^. To test the effects of Hsp33 on chemically denatured clients, CS from porcine heart (Sigma-Aldrich) was denatured to a final concentration of 12 μM in 6.0 M guanidinium-hydrochloride (GdmCl), 40 mM HEPES (pH 7.5) overnight at room temperature. To initiate aggregation of CS, the unfolded enzyme was diluted 1:160 into 1,600 μl 40 mM HEPES (pH 7.5) at either 20 or 30 °C in the absence or presence of Hsp33. To analyse Hsp33's effects on thermally unfolding CS, 0.15 μM CS were incubated in 1,600 μl 40 mM HEPES (pH 7.5) at the indicated temperatures in the absence or presence of Hsp33. For both assays, light scattering was monitored using a Hitachi F4500 fluorimeter equipped with a thermostated cell holder and stirrer. Excitation and emission wavelengths were set to 360 nm, and the excitation and emission slit widths were set to 2.5 nm. For competition studies between CS and NPY/NPY D4C IAM-TEMPO, a 10-fold molar excess of peptide to CS was used and the assay performed as described.

### ^19^F NMR experiments

Between 300–700 μM of each Hsp33^M172S^-tFPA variant was incubated in 40 mM KPi, 5 mM DTT, supplemented with 2 mM trifluoracetic acid for intensity and chemical shift referencing, and 10% (v/v) D_2_O. All ^19^F NMR experiments were performed on a 500 MHz Varian VNMRS spectrometer (Agilent Technologies), equipped with a 5 mm PFG OneNMR probe. ^19^F spectra were acquired using a single 90° pulse with carbon decoupling. ^19^F temperature-dependent spectra were acquired between 10 and 45 °C in steps of 5 °C. Peptide titration spectra of Hsp33 with NPY/NPY^D4C^-IAM-TEMPO were performed at 30 or 35 °C. Data were processed with iNMR.

### *In vitro* crosslinking procedure

Reaction mixtures containing 20 μM Hsp33_red_ or Hsp33_ox_ and 40 μM NPY or SNPY were crosslinked with either cyanur-biotin-dimercapto-propionyl-succinimide (CBDPS)-H8/D8 (Creative Molecules)^31^, ABAS-^12^C6/^13^C6 (Creative Molecules)^32^ or 1-ethyl-3-(3-dimethylaminopropyl)carbodiimide (EDC) (Sigma-Aldrich). To facilitate the identification of Hsp33-NPY zero-length crosslinked peptides (that is, EDC), 20 μM Hsp33 was prepared as an equimolar mixture of ^14^N- and ^15^N-metabolically labelled protein and incubated with 40 μM NPY.

For crosslinking with CBDPS, mixtures of wild-type Hsp33 and NPY were crosslinked by addition of 1 mM of an equimolar mixture of light and heavy isotopic forms of cyanur-biotin-dimercapto-propionyl-succinimide (CBDPS)-H8/D8 (Creative Molecules)^31^ dissolved in dimethyl sulfoxide and incubation at room temperature for 15 min. The crosslinking reaction was quenched by addition of 10 mM ammonium bicarbonate (ABC) (Sigma-Aldrich).

For crosslinking with ABAS, samples were incubated with 1 mM of an equimolar mixture of light and heavy isotopic forms of ABAS-^12^C6/^13^C6 (Creative Molecules)^32^ dissolved in dimethyl sulfoxide. To crosslink the proteins, the samples were exposed to UV irradiation from the top of an open 0.2 ml reaction tube with a 25 W 254 nm UV lamp (UVGL-58; 115 V, 60 Hz and 0.16A) (2 cm distance) for 10 min at room temperature. The crosslinking reaction was then quenched by addition of 10 mM ABC.

For crosslinking with EDC, samples were crosslinked by addition of 60 mM 1-ethyl-3-(3-dimethylaminopropyl)carbodiimide (EDC) (Sigma-Aldrich) at room temperature for 15 min. The crosslinking reaction was quenched by addition of 10 mM ABC.

### Proteolytic digest of *in vitro* crosslinking products

Crosslinked proteins were digested with either trypsin (Promega) for 18 h at 37 °C or proteinase K (Worthington Biochemical) for 120 min at 37 °C. Both proteases were used at a 1:20 protease to protein ratio. Trypsin and proteinase K digestions were inhibited by the addition of 10 mM 4-(2-aminoethyl)benzenesulfonyl fluoride hydrochloride (Sigma-Aldrich).

### Peptide enrichment and sample desalting

CBDPS crosslinked peptides were enriched on monomeric avidin beads (Pierce Biotechnology), eluted from the beads with a solution of 0.1% (v/v) trifluoroacetic acid (Pierce Biotechnology), 50% (v/v) acetonitrile (ACN) (Caledon Laboratories), lyophilized until dry, resuspended and reduced with 10 mM tris-(2-carboxyethyl)-phosphine (TCEP) (Pierce Biotechnology) for 10 min at room temperature, and finally acidified to pH 2 with formic acid (FA) (Thermo Fisher Scientific). Protease digested ABAS and EDC crosslinked samples were desalted using ZipTip C18 (Merck Millipore) pipette tips, lyophilized until dry, resuspended and reduced with 10 mM TCEP for 10 min at room temperature, and subsequently acidified to pH 2 with FA.

### LC/MS and data analysis of *in vitro* crosslinked peptides

Mass spectrometric (MS) analysis was carried out with an Easy-nLC II nano-HPLC system (LC) (Thermo Fisher Scientific) coupled to the ESI-source (Nanospray Flex Ion Source; Thermo Fisher Scientific) of an Orbitrap Velos Pro mass spectrometer (Thermo Fisher Scientific), as described earlier^49^. Samples were injected onto a 100 μm ID, 360 μm OD 1-cm trap column packed with Magic C18AQ (Bruker-Michrom), 100 Å, 5 μm pore size (prepared in-house) and desalted by washing for 15 min with 0.1% (v/v) FA. Peptides were separated with a 60 minute gradient (0–60 min: 4–40% B; 60–62 min: 40–80% B; 62–70 min: 80% B; with solvent B: 90% ACN, 10% water; and 0.1% FA) on a 75 μm ID, 360 μm OD 15 cm analytical column packed (in-house) with Magic C18AQ, 100 Å, 5 μm pore size with IntegraFrit (New Objective) and equilibrated with 95% solvent A (2% (v/v) ACN, 98% water and 0.1% (v/v) FA). Mass spectrometry data were acquired with Xcalibur (version 2.1.0.1140) in data-dependent MS/MS mode. Dynamic exclusion was set to 60 s with a repeat count of 2 and a repeat duration of 15 s. Mass spectrometry scans (*m/z* 400–2,000 range) and MS/MS scans were acquired at 60,000 and 30,000 resolution, respectively. MS/MS fragmentation was performed by collision-induced dissociation at normalized collision energy of 35%. Fourier transform mass spectrometry full automatic gain control target was 1,000,000 and Fourier transform mass spectrometry MSn automatic gain control target was 100,000. For CBDPS-H8/D8 and ABAS-^12^C6/^13^C6 crosslinked samples, mass differences between light and heavy isotopic forms of 8.05 and 6.02 Da were used in the Mass Tags setting, respectively^49^. For PICUP and EDC experiments involving ^15^N metabolically labelled Hsp33, a top 6 acquisition method was used. Proteome Discoverer (version 1.4.0.288) was used to generate.MGF files from.RAW files. Data analysis was performed using DXMSMS Match^50^ of ICC-CLASS^51^ and ^14^N^15^N DXMSMS Match^52^.

### ^1^H–^15^N HSQC protein NMR measurement

An amount of 300–500 μM Hsp33^Y12E^ was incubated in 40 mM KPi, 5 mM DTT, supplemented with 10% (v/v) D_2_O. All ^1^H–^15^N-NMR experiments were performed on an 800 MHz Varian VNMRS spectrometer (Agilent Technologies), equipped with a 5 mm HCN inverse probe with *z*-axis gradient. The NMR operating software was VnmrJ 3.2. Data were processed with iNMR.

## Additional information

**How to cite this article:** Groitl, B. *et al.* Protein unfolding as a switch from self-recognition to high-affinity client binding. *Nat. Commun.* 7:10357 doi: 10.1038/ncomms10357 (2016).

## Supplementary Material

Supplementary Information Supplementary figures 1-4 and Supplementary Tables 1-2.

Supplementary Data Mass spectrometric analysis of the in vitro crosslinking products between Hsp33ox / Hsp33red and neuropeptide Y.

## Figures and Tables

**Table 1:** Summary of identified Hsp33_ox_-NPY crosslinked peptides.

**Crosslinker**	**Protease**	Modified amino acid*	**Hsp33 peptide sequence**
ABAS (7 Å)	PK	44	E_35_NHDYPQPV***K***N_45_
	PK	**62**	L_61_***K***FDGD_66_
	PK	**62**	L_61_***K***FDGDIT_68_
	PK	**107**	G_98_EIPENADL***K***_107_
	PK	**198**	T_194_ETI***K***_198_
CBDPS (14 Å)	T	1	***M***_1_IMPQHDQLHR_11_
	T	12	***Y***_12_LFENFAVR_20_
	PK	**62**	L_61_***K***FD_64_
	PK	**107**	G_98_EIPENADL***K***_107_
	PK	166	G_165_***K***PA_168_
	PK	231	D_227_VEF***K***_231_
EDC (0 Å)	T	102	V_96_QGEIP***E***NADLK_107_
	T	150	S_149_***E***QLPTR_155_
	PK	**198**	T_196_I***K***TEELLTLPANEV_210_

ABAS, azido-benzoic-acid-succinimide; CBDPS, cyanur-biotin-dimercapto-propionyl-succinimide; EDC, (3-dimethylaminopropyl)carbo diimide; NPY, neuropeptide Y; PK, protease K; T, trypsin.

^*^Bold indicates residue was identified by at least two different crosslinkers. Bold and italicized alphabets indicate amino acids in Hsp33 peptides that were found crosslinked to NPY.
