# Supplementary material for: Protein unfolding as a switch from self-recognition to high-affinity client binding
Source: Nat Commun. 2016 Jan 20;7:10357. doi: 10.1038/ncomms10357 (PMC4735815; doi:10.1038/ncomms10357)
Supplement: Supplementary Information — Supplementary figures 1-4 and Supplementary Tables 1-2. [file ncomms10357-s1.pdf]

Supplementary Figure 1

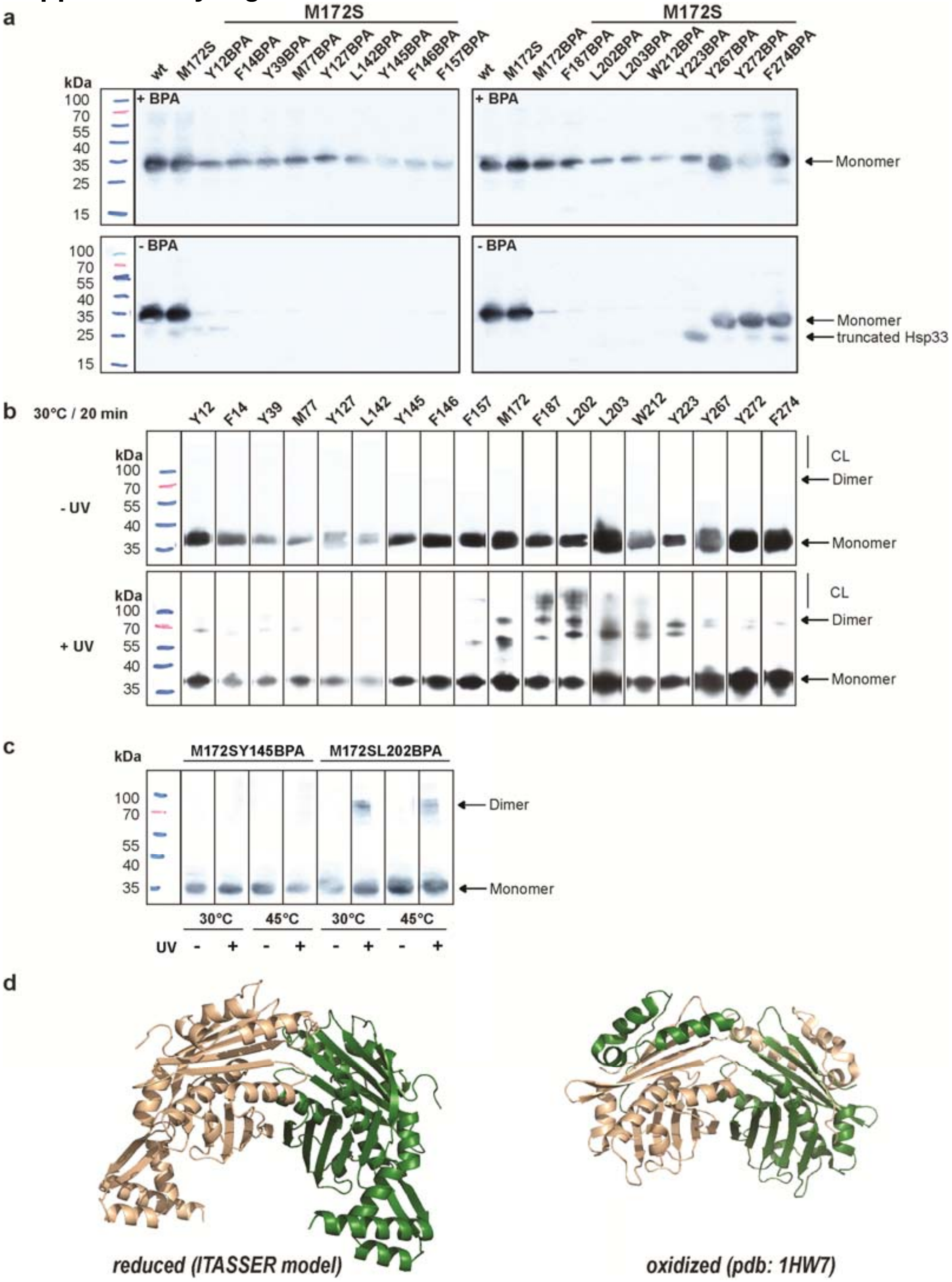

Supplementary Figure 1 *In vivo* crosslinking at 30 °C and crystal structures of Hsp33. (**a**) Expression of Hsp33^{M172SBPA} variants in the presence or absence of BPA (1 mM) as tested by western blot using anti-Hsp33 antibodies. Strains expressing Hsp33^{M172S} variants with amber mutations at position Y223, Y267, Y272, or F274 accumulate truncated versions of the respective Hsp33 variants in the absence of BPA. (**b**) Western blot analysis of the *in vivo* crosslinking products at 30 °C (for details see **Fig. 1b**). After incubation of *E. coli* cells overexpressing the Hsp33^{M172SBPA} variants at 30 °C, the cells were exposed to UV irradiation for 10 min to induce crosslinking (CL). Controls were left untreated (no UV irradiation). (**c**) Western blot analysis of purified Hsp33^{M172SY145BPA} (negative in *in vivo* crosslinking experiments) or Hsp33^{M172SL202BPA} (positive in *in vivo* crosslinking experiments) incubated at either 30°C or 45°C before and after UV-crosslinking. The 33 kDa monomer and the ~70 kDa Hsp33 dimer are indicated. No higher migrating bands were detected. (**d**) Cartoon depiction of I-TASSER model (left) and domain-swapped model (right) of Hsp33 (PDB 1HW7).

Supplementary Figure 2

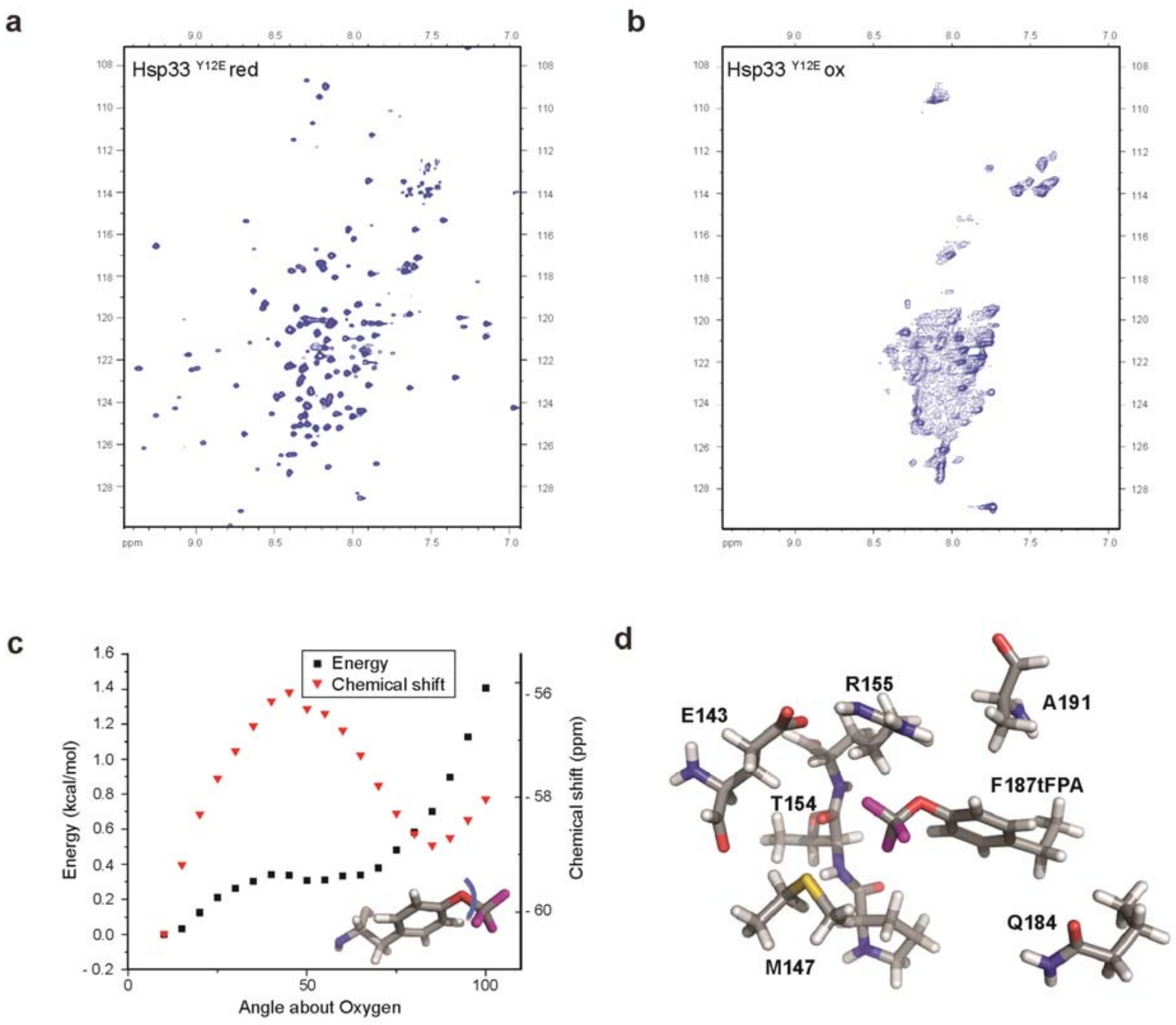

Supplementary Figure 2 NMR spectra of Hsp33^{Y12E} and Quantum Mechanics (QM) calculations. (**a**) ^{15}N 2D HSQC of reduced ^{15}N-labeled Hsp33^{Y12E}. This Hsp33 variant is known for its constitutively unfolded linker domain, which mediates full chaperone activity even in its reduced, zinc-coordinated form. It can therefore be considered to be the least unfolded variant of Hsp33 that shows full chaperone function. Although well dispersed, only one third of the expected peaks are well defined. (**b**) ^{15}N 2D HSQC of ^{15}N-labeled oxidized Hsp33^{Y12E}. (**c**) Computational dihedral scan of tFPA chemical shift and conformational energy. The large chemical shift change combined with the low rotational barrier of the trifluoro group results in a high sensitivity of tFPA to binding or conformational changes. (**d**) QM model of F187tFPA substitution in the closed state. The adjacent positively charged R155 side chain causes a down-field change in the calculated ^{19}F chemical shift of tFPA.

Supplementary Figure 2

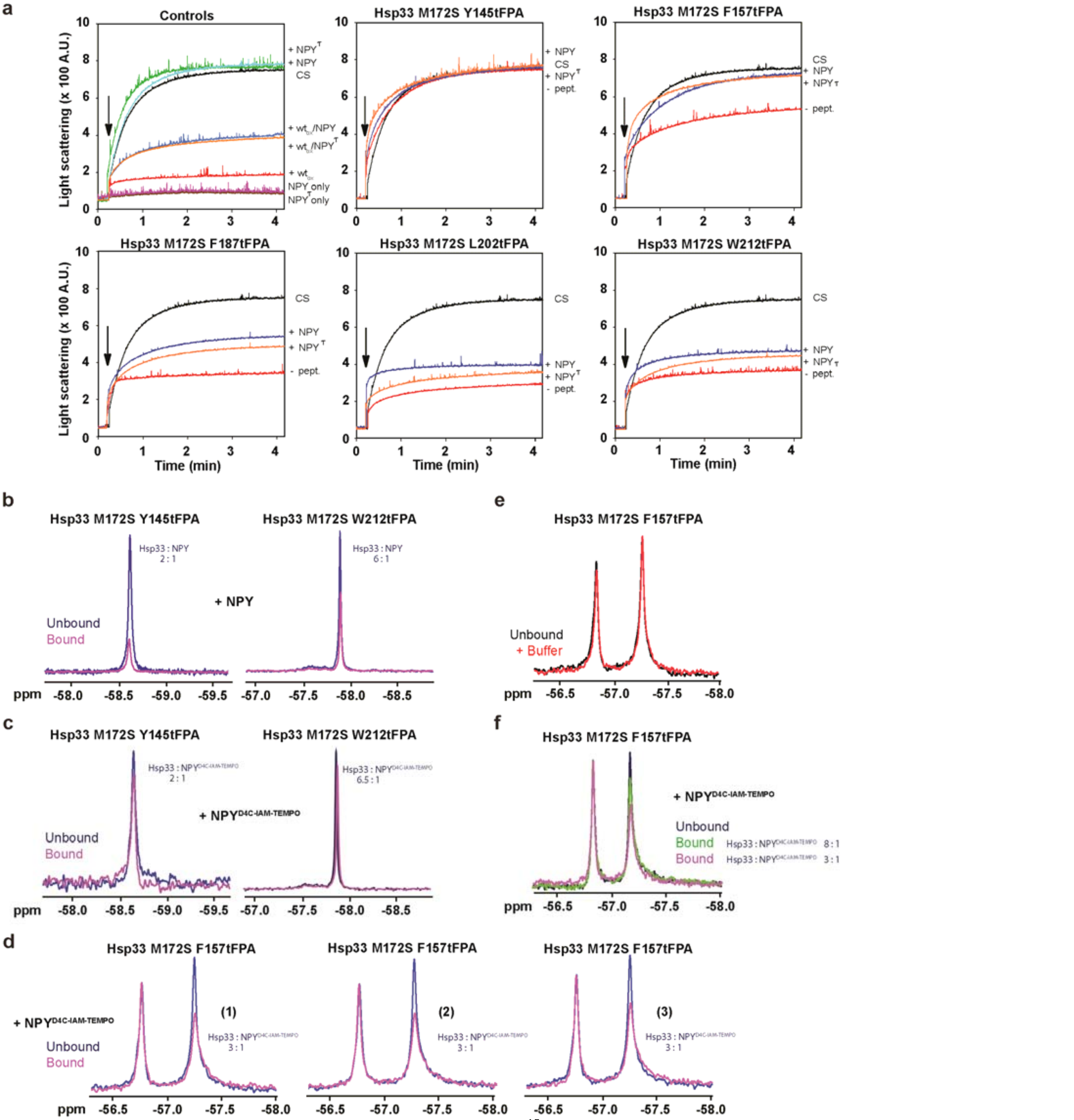

Supplementary Figure 3 Peptide competition assays and ^{19}F NMR spectra. (**a**) The influence of a four-fold molar excess of oxidized, wild-type Hsp33 and reduced Hsp33^{M172StFPA} variants on the aggregation of chemically unfolded CS (150 nM) was monitored at 30 °C in the presence or absence of a ten-fold excess of NPY or NPY$^{D4C\text{-}IAM\text{-}TEMPO}$ (labeled NPY^{T} here) to CS. A loss in the prevention of aggregation of CS upon peptide addition indicates binding of NPY or NPY$^{D4C\text{-}IAM\text{-}TEMPO}$ to Hsp33. (**b**) Binding of NPY or (**c**) NPY$^{D4C\text{-}IAM\text{-}TEMPO}$ to select Hsp33^{M172StFPA} variants as monitored by ^{19}F NMR experiments. The decreased intensity of Hsp33^{M172SY145tFPA} shown in (**b**) is due to aggregation of the protein sample. The lack of decreasing peak intensity upon addition of the paramagnetic TEMPO tag shown in (**c**) demonstrates that binding does not occur at these sites. (**d**) Reproducibility of peptide-dependent line broadening of the active state resonances as shown by using technical replicates of NPY$^{D4C\text{-}IAM\text{-}TEMPO}$ addition to Hsp33^{M172SF157tFPA}. (**e**) No substantial chemical shift changes were observed upon addition of NPY$^{D4C\text{-}IAM\text{-}TEMPO}$ buffer to Hsp33^{M172SF157tFPA}. (**f**) NMR titration experiment of increasing amounts of NPY$^{D4C\text{-}IAM\text{-}TEMPO}$ to Hsp33^{M172SF157tFPA} illustrates a concentration-dependent loss in peak intensity.

Supplementary Figure 4

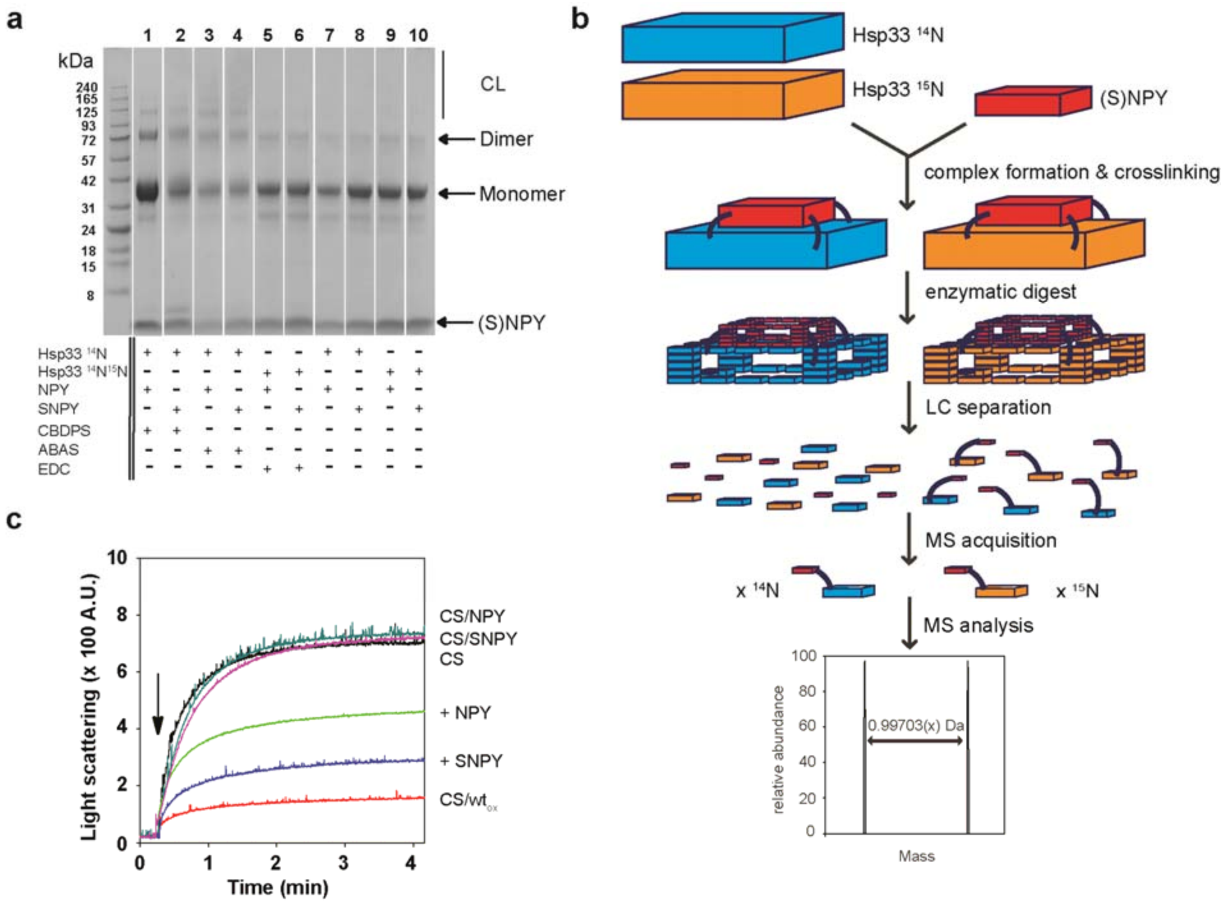

Supplementary Figure 4 *In vitro* crosslinking procedure and scheme. (**a**) SDS-PAGE gel of *in vitro* crosslinking conditions of Hsp33 and NPY or SNPY, which carries one additional Ser residue at the N-terminus. (**b**) Scheme of the experimental procedure employed for the *in vitro* crosslinking experiments with EDC. Experiments with isotopically labeled CBDPS and ABAS require only ^{N}14-labeled Hsp33. (**c**) Competition assays of NPY or SNPY. The influence of a four-fold molar excess of reduced, zinc-reconstituted as well as HOCl oxidized wild-type Hsp33 on the aggregation of chemically unfolded CS (150 nM) was monitored at 30 °C in the presence or absence of a ten-fold excess of NPY or SNPY.

Supplementary Table 1 Positive and negative interaction sites in activated Hsp33$_{ox}$

Hsp33 residues	Limited proteolysis[16]	*In vivo* crosslinking	*In vitro* crosslinking	Interactions ^{19}F NMR
M1			++	
R11	-			
Y12		++	++	
F14		-		
R20	-			
Y39		++		
K44	++		++	
K62	++		++	
M77		-		
R91	-			
R95	-			
E102			+++	
K107			++	
R126	-			
Y127		-		
L142		-		
Y145		-		-
F146		-		
R148	++			
E150			+++	
R155	-			
F157		++		+++
R159	++			
K166			++	
M172		++		
F187		+++		++
K198	++		+++	
L202		+++		+
L203		++		
W212		(+)		-
R213	-			
Y223		++		
K231	-		++	
Y267		-		
Y272		-		
F274		-		

Cell colors correspond to color scheme in **Figure 5**.

Supplementary Table 2 Strains and plasmids used in this study

	Marker	Relevant genotype	Source
**Strains**			
BL21(DE3) *hslO*⁻	Kan^R	F^- *ompT gal dcm lon hsdSB* (*rB*⁻ *mB*⁻)λ (DE3 (*lacI lacUV5-T7 gene 1 ind1 sam7 nin5*)) *hslO*::kan	[40]
NEB10β		Δ*(ara-leu) 7697 araD139 fhuA* Δ*lacX74 galK16 galE15 e14-* ϕ80d*lacZ*Δ*M15 recA1 relA1 endA1 nupG rpsL* (Str^R) *rph spoT1* Δ*(mrr-hsdRMS-mcrBC)*	New England Biolabs
**Plasmids**			
pEVOL	Cm^R	Plasmid used for incorporation of BPA	[19]
pDULE2-pCNF	$Spec^R$	Plasmid used for incorporation of tFPA	[27]
pET11a	Amp^R	IPTG inducible expression vector	[17]
pET21b	Amp^R	IPTG inducible expression vector with C-terminal His_6	Novagen
pET11a *hslO*	Amp^R	Plasmid expressing wild-type Hsp33 (*hslO*)	[17]
pET11a *hslO* M172S	Amp^R	Plasmid expressing Hsp33 M172S	[17]
pET21b *hslO* M172S	Amp^R	*hslO M172S* cloned into NdeI/HindIII of pET21b	This study
pBG31	Amp^R	$Y12^{UAG}$ mutation in pET11a *hslO* M172S	This study
pBG32	Amp^R	$F14^{UAG}$ mutation in pET11a *hslO* M172S	This study
pBG33	Amp^R	$Y39^{UAG}$ mutation in pET11a *hslO* M172S	This study
pBG34	Amp^R	$M77^{UAG}$ mutation in pET11a *hslO* M172S	This study
pBG35	Amp^R	$Y127^{UAG}$ mutation in pET11a *hslO* M172S	This study
pBG36	Amp^R	$L142^{UAG}$ mutation in pET11a *hslO* M172S	This study
pBG37	Amp^R	$Y145^{UAG}$ mutation in pET11a *hslO* M172S	This study
pBG38	Amp^R	$F146^{UAG}$ mutation in pET11a *hslO* M172S	This study
pBG39	Amp^R	$F157^{UAG}$ mutation in pET11a *hslO* M172S	This study
pBG40	Amp^R	$M172^{UAG}$ mutation in pET11a *hslO* M172S	This study
pBG41	Amp^R	$F187^{UAG}$ mutation in pET11a *hslO* M172S	This study
pBG42	Amp^R	$L202^{UAG}$ mutation in pET11a *hslO* M172S	This study
pBG43	Amp^R	$L203^{UAG}$ mutation in pET11a *hslO* M172S	This study
pBG44	Amp^R	$W212^{UAG}$ mutation in pET11a *hslO* M172S	This study
pBG45	Amp^R	$Y223^{UAG}$ mutation in pET11a *hslO* M172S	This study
pBG46	Amp^R	$Y267^{UAG}$ mutation in pET11a *hslO* M172S	This study
pBG47	Amp^R	$Y272^{UAG}$ mutation in pET11a *hslO* M172S	This study
pBG48	Amp^R	$F274^{UAG}$ mutation in pET11a *hslO* M172S	This study
pBG49	Amp^R	*hslO M172S* $Y12^{UAG}$ cloned into NdeI/HindIII of pET21b	This study
pBG50	Amp^R	*hslO M172S* $F14^{UAG}$ cloned into NdeI/HindIII of pET21b	This study
pBG51	Amp^R	*hslO M172S* $Y39^{UAG}$ cloned into NdeI/HindIII of pET21b	This study
pBG52	Amp^R	*hslO M172S* $M77^{UAG}$ cloned into NdeI/HindIII of pET21b	This study
pBG53	Amp^R	*hslO M172S* $Y122^{UAG}$ cloned into NdeI/HindIII of pET21b	This study
pBG54	Amp^R	*hslO M172S* $L142^{UAG}$ cloned into NdeI/HindIII of pET21b	This study
pBG55	Amp^R	*hslO M172S* $Y145^{UAG}$ cloned into NdeI/HindIII of pET21b	This study

pBG56	Amp^R	*hslO M172S F146*^{UAG} cloned into NdeI/HindIII of pET21b	This study
pBG57	Amp^R	*hslO M172S F157*^{UAG} cloned into NdeI/HindIII of pET21b	This study
pBG58	Amp^R	*hslO M172*^{UAG} cloned into NdeI/HindIII of pET21b	This study
pBG59	Amp^R	*hslO M172S F187*^{UAG} cloned into NdeI/HindIII of pET21b	This study
pBG60	Amp^R	*hslO M172S L202*^{UAG} cloned into NdeI/HindIII of pET21b	This study
pBG61	Amp^R	*hslO M172S L203*^{UAG} cloned into NdeI/HindIII of pET21b	This study
pBG62	Amp^R	*hslO M172S W212*^{UAG} cloned into NdeI/HindIII ofpET21b	This study
pBG63	Amp^R	*hslO M172S Y223*^{UAG} cloned into NdeI/HindIII of pET21b	This study
pBG64	Amp^R	*hslO M172S Y267*^{UAG} cloned into NdeI/HindIII of pET21b	This study
pBG65	Amp^R	*hslO M172S Y272*^{UAG} cloned into NdeI/HindIII of pET21b	This study
pBG66	Amp^R	*hslO M172S F274*^{UAG} cloned into NdeI/HindIII of pET21b	This study
